# Surgical Techniques for Managing Post-prostatectomy Erectile Dysfunction

**DOI:** 10.1007/s11934-017-0735-2

**Published:** 2017-09-30

**Authors:** Fabio Castiglione, David J. Ralph, Asif Muneer

**Affiliations:** 1 Department of Andrology, University College London Hospital, 250 Euston Road, London, NW1 2PG UK; 2 NIHR Biomedical Research Centre, University College London Hospital, London, UK

**Keywords:** Erectile function after radical prostatectomy, Nerve-sparing radical prostatectomy, Radical prostatectomy outcome

## Abstract

**Purpose of Review:**

Due to the increasing numbers of radical prostatectomies (RP) performed for prostate cancer, a substantial number of patients are now suffering from post-operative erectile dysfunction (ED). The aim of this study is to summarize the current literature on surgical techniques for managing post-prostatectomy erectile dysfunction.

**Recent Findings:**

The PubMed database was searched for English-language articles published up to Jan 2017 using the following search terms: “prostatectomy AND erectile dysfunction”, “prostatectomy AND penile prostheses”, and “prostatectomy AND penile implants”. All of the studies that evaluated medical treatment were excluded. In the last few decades, the understanding of the anatomy of the male pelvis and prostate has improved. This has led to significant changes in the nerve-sparing radical prostatectomy techniques, with the aim of preserving post-surgical erectile function (EF). In this scenario, the prostate vascular supply and the anatomy of the neurovascular bundles have a central role. Penile prosthesis implantation is considered the third-line treatment option for RP ED patients, and they have been reported to be a very successful treatment with the highest patient satisfaction rate.

**Summary:**

Considering the failure of penile rehabilitation, and the lack of evidence for accessory pudendal artery (APA) preservation and nerve graft, nerve-sparing surgery and penile prostheses represent, today, the only methods to permanently and definitively preserve or erectile function after RP.

## Introduction

The evolution of prostate cancer management has now taken a paradoxical turn. Large longitudinal studies investigating the role of active surveillance have made it clear that more than 40 to 50% of prostate cancers detected in the prostate-specific antigen (PSA) era are indolent [1]. Despite these findings, thousands of asymptomatic men suffering from a potentially harmless disease still undergo aggressive surgical treatment, and as a result, prostate cancer survivors are at risk of iatrogenic erectile dysfunction (ED). It has been postulated that only 10 to 23% of men aged below 60 years regain their baseline potency after bilateral nerve-sparing radical prostatectomy (NSRP) [2–4]. The use of robot-assisted radical prostatectomy (RARP) has not significantly reduced these post-operative ED rates [5]. A recent prospective trial comparing RARP to open retropubic radical prostatectomy (RRP) showed that only 29.6% of patients did not suffer from ED at 1 year following open RRP compared with 25.3% after RARP, despite RARP patients having a better performance status and more likely to undergo a nerve-sparing procedure [5]. Surprisingly, these rates from a real-life setting are tempering the initial enthusiasm about the functional outcomes of anatomical radical prostatectomy that has been growing over the past two decades.

This scenario is further complicated by the widespread use of various penile rehabilitation protocols, despite this being short of a solid evidence base [6]. In a survey conducted by the International Society for Sexual Medicine (ISSM) members from 41 countries, over 95% routinely prescribed phosphodiesterase type 5 inhibitors (PDE5i) to their prostatectomy patients, as part of a penile rehabilitation protocol [7]. For patients wishing to maintain the ability to have sexual intercourse and in whom pharmacological options have failed, end-stage treatment using penile prostheses surgery is an option.

The aim of the present study was to review the current surgical treatments for ED after radical prostatectomy.

## Methods

The PubMed database was searched for English-language articles published up to Jan 2017 using the following search terms: “prostatectomy AND erectile dysfunction”, “prostatectomy AND penile prostheses”, and “prostatectomy AND penile implants”. This review is primarily focused on the literature published within the past 10 years, although older papers closely related to this topic are also included. The present review evaluated only selected studies, evaluating the surgical therapy for erectile dysfunction after radical prostatectomy. All of the studies that evaluated medical treatment were excluded.

## Erectile Dysfunction After Radical Prostatectomy: How Big Is the Problem?

In the last decades, several studies have investigated the incidence of ED following RP with different and sometimes conflicting results. Factors such as the different classification and evaluation of EF, patient selection criteria, the surgical technique used and the different penile rehabilitation treatment account for this wide variability. EF recovery rates after an open radical prostatectomy have been reported to range from 31 to 86% [8]. Equally, potency rates for laparoscopic RP (LRP) have been showed to range from 42 to 76% [9].

More recently, Ficarra et al. published a meta-analysis of a RARP series and reported potency recovery rates of 32 to 68%, 50 to 86%, 54 to 90% and 63 to 94% at 3, 6, 12 and 24 months after surgery, respectively [10••]. The study analysed 15 case series, 6 studies comparing different techniques in the context of RARP, 6 studies comparing RARP with RRP and 4 studies comparing RARP with LRP [10••].

## Pathophysiology of Post-prostatectomy ED: Is It a Neuronal Problem?

Penile erection is based on neurovascular pathways orchestrated by psychological, emotional and hormonal factors where the interplay both neuronal and vascular components are crucial and indispensable. A penile erection requires neurotransmitters, such as nitric oxide (NO) and Prostaglandins, released by the cavernous nerve (CN) terminals, which provide parasympathetic innervation to the corpora cavernosa. These neurotransmitters induce the relaxation of the smooth muscle of the arteries and of the cavernosal smooth muscle [11].

The CN terminals arise from the pelvic plexus that is located within the fibro-fatty plane between the bladder and the rectum, and they run in close vicinity to the tips of the seminal vesicles along the dorsolateral aspect of the prostate, between the capsules of the prostate. These fibers run together with blood vessels and comprise the neurovascular bundles (NVBs) as described by Walsh et al. [12, 13•, 14, 15].

Erectile dysfunction after non-nerve-sparing radical prostatectomy is an inevitable consequence of the deliberate or unwanted transection of the CN.

In contrast, erectile dysfunction after nerve-sparing radical prostatectomy (NSRP) is a complex mechanism and still not completely understood. However, it is now clear that age comorbidities such as pre-operative erectile function are independent prognostic factors for the recovery of erectile function following surgery [16–18].

A few days after nerve-sparing surgery, ED is a result of iatrogenic functional damage (traction injury, diathermy) to the periprostatic neurovascular bundle, which results in neuropraxia which is evident immediately after prostatectomy with loss of nocturnal and sexually stimulated erections.

Urologists and patients may note moderate erectile activity shortly after surgery, such as at the time of catheter manipulation or removal. These events may bring about high hopes for recovery of erectile function, but the nerves are partially conductive until the ensuing Wallerian degeneration has set in several days following the neuropraxia. The resulting denervation of the corpora cavernosa results in a decrease in the rate and quality of early morning and nocturnal erections, and thus promotes persistent cavernous hypoxia, as oxygenated blood is mainly supplied during erectile activity [19–21]. Both in vitro and in vivo studies support the theory that penile hypoxia results in collagen accumulation, smooth-muscle apoptosis and ultimately cavernosal fibrosis. Finally, these changes within the corpus cavernosum contribute to venous leakage and permanent ED, even if the normal function of the nerves return [22].

It has been postulated that another mechanism involved in post-prostatectomy ED may be the injury of the accessory pudendal arteries [23], which have been described in up to 75% of patients, and could lead to the development of penile hypoxia [23].

Finally, recent results from Salamanca’s group [23] have shown that neurogenic contractile responses (sympathetic) are increased in the corpus cavernosum (CC) of rats following cavernous nerve injury and in cavernosal tissues from men suffering with post-RP ED. This is simultaneous to a profound impairment of neurogenic relaxant responses that result in a marked imbalance in the neurogenic control of penile smooth muscle tone favouring contractile input over relaxant drive, thus antagonizing the erectile process. The author concluded that alpha-adrenergic modulation, especially selective **α**1A-blockade, improves erectile and cavernosal functions after BCNI [24••].

## Surgical Management of Erectile Dysfunction

In the last couple of decades, the anatomy of the male pelvis and prostate has improved. This has led to significant changes in the nerve-sparing radical prostatectomy techniques, with the aim of preserving post-surgical erectile function (EF). In this scenario, the prostate vascular supply and the anatomy of the NVBs have a central role [13•].

## Prostate Arterial Supply: Artery-Sparing Surgery Myth or Reality?

In 35 to 56% of cases, the prostate arteries arise from the internal pudendal artery [24••]. The common gluteal pudendal trunk is the next most frequent origin (15–28%), followed by the obturator artery and the inferior gluteal artery [13•]. Two principal bifurcations of the prostatic artery can be identified as follows: a posterior pedicle and an anterior pedicle at the level of the lateral side of the prostate, reaching the prostate apex. At this level, the anterior capsular prostate branches are responsible for the ancillary penile blood supply [13•]. The intraoperative preservation of these structures may be responsible for preservation of post-surgical EF.

The penile arterial blood supply is derived exclusively from the internal pudendal artery (IPA) or from the IPA and an anatomical variant known as the accessory pudendal artery (APA) or from the APA alone [23, 25–27].

The APA is defined as any artery located in the periprostatic region running parallel to the dorsal vascular complex and extending caudal toward the anterior perineum. A recent meta-analysis, [28] evaluating 23 studies, reported that the APA was present in 28.5% of patients. When present, unilateral accessory pudendal arteries were most common (pooled prevalence estimate 72.5%), or they were present on the right or the left side (pooled prevalence estimate 48.0 or 52.0%, respectively). They were most commonly originating from the obturator artery and the inferior vesical artery (pooled prevalence estimate 48.9 and 29.6%, respectively). The most common type was the apical accessory pudendal arteries (pooled prevalence estimate 60.9%).

Numerous reports have shown that an injury to the APA can induce vasculogenic ED. Conversely, Box et al. recently evaluated the consequence of damaging the APA in 200 patients treated with RARP; they reported that 19 patients had an injury of the APA. Surprisingly, 18 of them reported post-prostatectomy EF recovery [29].

More evidences are required to assess whether APA preservation can improve the EF outcome after radical prostatectomy.

## Neurovascular Bundles: the Truth Is Between the Layers

The neuronal supply to the corpora cavernosa originates from the pelvic plexus and transgresses the lateral aspect of the bladder neck passing posterolaterally to the tip of seminal vesicles [13•]; based on this anatomical aspect, it can be postulated that the preservation of the tip of the seminal vesicles may aid the recovery rate for EF. However, no scientific evidence is available for this. A prospective controlled study, including 52 patients (followed up for 6 months), showed that a seminal vesicle tip-sparing technique may preserve pelvic innervation and reduce urinary incontinence. No data on EF recovery rates were reported in this study [12]. Furthermore randomized studies are necessary to evaluate the impact of seminal vesicle-sparing radical prostatectomy on restoration of urinary continence.

NVB fibers are contained in a multi-layered fascia that can be either fused or separated from the prostatic capsule; the relationship between this fascia, known as the periprostatic fascia (PPF) and the NVB, has been extensively investigated with the intent to develop a NS technique able to preserve the largest number of cavernous fibers [13•]. The multi-layered character of the PPF allows several degrees of dissection with the intent of leaving a more or less thick tissue layer on the prostate as a safety margin. This anatomical characteristic lets the surgeon, despite individual patient variation, to choose the degree of NS dissection based on the risk of extraprostatic extension of the tumour [13•].

In the past, three different dissection planes had been described: an intrafascial, interfascial and extrafascial plane (Table 1) [30]. Successively, a new classification was described by Montorsi et al. proposing “full, partial and minimal” NS techniques as matching to intrafascial, interfascial and “sub” extrafascial dissections, respectively (Table 1) [31].

**Table 1:** Neurovascular bundles: nerve-sparing technique

Author	Classification	Description
Waltz et al. 2010 [30]	Intrafascial dissection	Intrafascial dissection of the NVB is considered a dissection that follows a plane on the prostate capsule, remaining medial or internal to the PF at the anterolateral and posterolateral aspect of the prostate and also remaining anterior to PPF/SVF
	Interfascial dissection	Interfascial dissection of the NVB is considered a dissection outside or lateral to the PF at the anterolateral and posterolateral aspects of the prostate combined with a dissection medial to the NVB at the 5-o’clock and 7-o’clock positions or the 2-o’clock and 10-o’clock positions of the prostate in axial section
	Extrafascial dissection	Extrafascial dissection of the NVB (non-NS) is considered a dissection-carried lateral to the LAF and posterior to the PPF/SVF. In this case, the NVB running along the posterolateral aspect of the prostate is completely resected with LAF, PF, and PPF/SVF remaining on the prostate
Montorsi et al. 2012 [31]	Full	Full dissection of the NVB is considered a dissection between the prostatic capsule and the multilayer tissue of the prostatic fascia
	Partial	Partial dissection of the NVB is considered a dissection within the multilayer tissue of prostatic fascia
	Minimal	Minimal dissection of the NVB is considered a dissection aimed to preserve only the cavernous nerves running at the posterolateral surface of the prostate
Tewari et al. 2011 [32]	Grade 1	Grade 1 dissection of the NVB is considered a dissection of the Denonvilliers’ and lateral pelvic fascia (LPF) just outside the prostatic capsule.
	Grade 2	Grade 2 dissection of the NVB is considered a dissection through the Denonvilliers’ (leaving deeper layers on the rectum), and LPF is taken just outside the layer of veins of the prostate capsule
	Grade 3	Grade 3 dissection of the NVB is considered a dissection through the outer compartment of the LPF (leaving some yellow adipose and neural tissue on the specimen), excising all layers of Denonvilliers’ fascia
	Grade 4	Grade 4 dissection of the NVB (non-NS) is considered a dissection of the LPF and Denonvilliers’ fascia containing most of the periprostatic neurovascular tissue
Schatloff et al. 2012 [33]	Grade 5	Grade 5 (≥ 95%) dissection of the NVB is considered a dissection performed medial to LA just outside the prostatic fascia at the pearly areolar tissue between the prostate and the NVB
	Grade 4	Grade 4 (75%) dissection of the NVB is considered a dissection performed using a sharp dissection at a plane between the LA and the prostatic capsule across the NVB—not at the pearly areolar plane, as in the case of a complete NS
	Grade 3	Grade 3 (50%) dissection of the NVB is considered a dissection at the artery’s lateral aspect; therefore, the LA is clipped at the level of the prostatic pedicle
	Grade 2	Grade 2 (< 50%) dissection of the NVB is considered a dissection performed several millimeters lateral to the artery, following the prostatic contour
	Grade 1	Grade 1 (0%) dissection of the NVB (non-NS) is considered a dissection performed by sequentially clipping below the prostate across the NVB. The prostate is lifted up and only rotated when approximating the apex as the pelvis gets narrow and the NVB curves in the direction of the perineum

In 2011, Tewari et al. reported a new 4-degree NS approach [32], taking as a landmark the veins located on the lateral aspects of the prostate. In this retrospective study, 2317 patients were evaluated. All the patients had robotic prostatectomy by a single surgeon at a single institution between January 2005 and June 2010 [32]. The author showed that there was a significant difference across different NS grades in terms of the percentages of patients who had intercourse and returned to baseline sexual function (*P* < 0.001), with those that underwent NS grade 1 having the highest rates (90.9 and 81.7%) as compared to NS grades 2 (81.4 and74.3%), 3 (73.5 and 66.1%) and 4 (62 and 54.5%) (Table 1) [32].

Schatloff et al. [33] described a 5-degree dissection using as a landmark the artery (LA) running along the lateral aspect of the prostate, on the medial aspect of the NVB. The LA can be recognized, intraoperatively, in up to 73% of cases. The authors demonstrated that a higher NSS was significantly correlated with a decreasing area of residual nerve tissue on prostatectomy specimens (*P* < 0.001); no data on erectile function were reported (Table 1) [33].

Currently, no consensus exists regarding the possibility of a higher incidence of post-operative ED associated with more extended PLNDs.

## Past vs Future, Open vs Robotic: Is There a Real Difference Between the Techniques?

Despite the number of studies available in the literature, the comparison of EF outcomes between open and minimally invasive surgery (laparoscopic and robotic techniques) is very difficult to perform owing to a lack of standardized methodology and because a lot of the evidence is derived from retrospective studies. Ficarra et al. in a meta-analysis on RP series up to 2012, including a cumulative analysis of records from open radical prostatectomy (ORP) versus RARP series, showed a significant benefit in favour of RARP, with a risk reduction for ED of 23.6% [10••].

However, in the last decades, several prospective randomized clinical trials were published. Tewari et al. in 2003 reported the results of a single-institution, prospective and unrandomized clinical trial including 100 patients undergoing ORP and 200 undergoing RARP. After RARP, patients achieved continence and return of erections more quickly than after ORP (160 vs 44 and 180 vs 440 days, both *P* < 0.5). The median return to intercourse was 340 days after VIP, but after RRP, half of the patients have yet to resume intercourse at 700 days (*P* < 0.05) [34].

Similarly, Haglind et al. showed, at 12 months following surgery, a significant benefit in favour of RARP, in a multicentre prospective unrandomized study including 778 ORP patients and 1847 RARP patients [5].

More recently, a sub-analysis of the REACTT trial conducted to compare once-daily tadalafil, on-demand tadalafil and placebo for penile rehabilitation showed that the odds of achieving EF recovery at the end of the drug-free washout period were twice as high for RARP compared to ORP, but no difference was reported between LRP and ORP patients. The same results were also published by Magheli et al. showing, in a comparative analysis between LRP and ORP, no difference between the two groups in terms of post-operative EF [35].

Few studies have compared the sexual outcome between LPR and RARP with conflicting results. Ficarra et al. showed, in a meta-analysis [36], no statistical difference when comparing EF recovery rate between LRP and RARP. These results are in contrast with a recent prospective randomized study conducted on 128 patients treated with either LRP or RARP using a BNS approach. In this study, RARP patients exhibited a higher rate of return to baseline IIEF-EF scores than LRP patients.

Taken together, this data shows a benefit in terms of EF recovery for RARP patients in comparison to those treated with laparoscopic techniques or open surgery; however, recently published large population-based studies comparing ORP and RARP have reported conflicting data [37–39].

## Penile Prosthesis: Is it the Best Solution?

According to the EAU guidelines on Male Sexual Dysfunction [40], penile prosthesis implantation is considered the third-line treatment option for ED patients after first- and second-line pharmacological options have been exhausted.

Currently, there are two types of penile prostheses: inflatable and malleable types. The inflatable penile prostheses can be subdivided into two- and three-piece devices. IPP is considered a superior option compared to a malleable prosthesis as it produces penile rigidity and flaccidity that closely replicates a normal penile erectile function and is easier to conceal [41].

In the RP scenario, patients undergoing NNS or NS surgery for PCa may benefit from penile prosthesis surgery after the failure of other treatment options, and it has also been shown to be safe in patients who have undergone RP or RT for PCa [40].

Penile prostheses have been reported to be a very successful treatment with the highest patient satisfaction rate, up to 90% [41–44].

The impact of penile prosthesis surgery extends beyond sustaining penile rigidity as it also includes favourable psychosocial outcomes. Recently, Bettocchi et al. have reported greater partner satisfaction rate after penile implant procedure [45].

Penile prosthesis surgery does not affect the continence recovery rates after RP although a small study has shown an improvement [46]. In addition, implantation of a penile prosthesis does not preclude later artificial sphincter or urethral sling surgery. Moreover, the two procedures may also be performed simultaneously [47, 48].

Despite excellent patient and partner satisfaction rates and safety profiles, penile prostheses appear to be underused in the RRP group [49].

In 2005 for instance, Stephenson et al. [50] reported data from the Surveillance Epidemiology and End Results cancer registry, showing that only 1.9% of patients treated with either RP or RT underwent penile prosthesis surgery. More recently, Tal et al. [49] showed a lower utilization rate in a SEER population comprising 68,558 subjects, including 52,747 who had RT and 15,811 who had RP as primary prostate cancer treatment. The penile implant utilization rate was 0.8% for the entire group, 0.3% for the RT group and 2.3% for the RP group. Younger age, and African American or Hispanic race, initial treatment modality and being unmarried are the most important predictors of penile implant utilization [49].

## Nerve Graft After Radical Prostatectomy

Despite the advance in NS techniques, the resection of the NVB is still sometimes necessary to accomplish an adequate cancer-free margin. Additionally, iatrogenic injury to the prostatic nerves may occur during RP irrespective of the technique used.

Several basic science studies in rats have shown that the cavernous nerves can be replaced with nerve grafts with the intent to restore EF [50–53]. Once again, creative researchers and surgeons started thinking about the possibilities and tested this modality in the clinical setting. Immediate interpositional nerve grafting, using autologous sural or genitofemoral nerves, of the prostatic plexus can be performed for ameliorating post-RP urinary continence and EF after nerve resection or damage [53–63]. Although different studies have demonstrated the feasibility of immediate nerve reconstruction after RP, clinical outcomes have been variable, and few studies have examined patients with long-term follow-up or in comparison with control groups [55–64]. Furthermore, results of a well-designed randomized controlled trial demonstrated no benefit of unilateral nerve grafting after prostatectomy [54]. As a result, these developments greatly tempered the enthusiasm for cavernous nerve reconstruction, and nerve grafting has now been largely abandoned and it is now considered an experimental procedure.

However, in the last decades, a new Bio-graft has been tested with promising results.

Patel et al. [64] recently showed that dehydrated human amnion/chorion membrane allograft nerve wrap around the NVB accelerates early return to continence and potency following robot-assisted radical prostatectomy.

## New Perspective

### Stem Cell

Stem cells (SCs) are defined by their self-renewal capability and differentiation potential to other cell lines. These characteristics are responsible for the maintenance of the SC population and the potential for tissue or organ regeneration. SCs are classified according to their differential potential in totipotent, pluripotent, multipotent, progenitor or precursor cells [65, 66].

Embryonal SCs (ESC) are pluripotent SCs. They can differentiate into cells from all germinal layers. Multipotent SCs can be isolated from different adult tissues and differentiate into any cell type within their own germinal layer.

Adult mesenchymal stem cells (MSCs) are classified as multipotent stem cells which are able to differentiate into several subtypes of mesenchymal cells. Until recently, this property had only been studied for its direct medical implications and therapeutic uses [67, 68]. However, MSCs release a wide spectrum of regulatory and trophic factors (growth factors, cytokines and chemokines). This suggests a paracrine effect as “site-regulated drugstores” in vivo and can influence tissue even if they do not engraft or differentiate [69–72]. In the last few decades, researchers have tried applying SCs by exploiting their immune-regulatory properties to several therapeutic scenarios [69]. A few examples include graft-versus-host disease (GVHD) in bone marrow transplantation, multiple sclerosis, brain and spinal cord injury, arthritis, myocardial infarction and Crohn’s disease. Not surprisingly, SC treatment is also one of the up-and-coming curative treatment options for ED [69–72].

In 2004, Bochinski et al. [73] first tested the injection of green fluorescent protein-labelled ESC either into the corpus cavernosum or to the major pelvic ganglion (MPG) in the rat model of radical prostatectomy with cavernous nerve injury (CNI). Erectile function improved significantly in the treated animals compared to the controls [73].

The efficacy of bone marrow SCs (BMSCs) was tested by Kendirci et al. [74]. In this study intracavernosal BMSCs injection were performed in rats after CNI. Erectile response to cavernous nerve stimulation (CNS) was partially restored at 4 weeks [74].

Albersen et al. [75] showed that the administration of ADSC-derived lysate in the CNI rat model showed improved responses to CNS compared with rats injected with adipose-derived stem cells (ADSCs). Interestingly, both ADSC and lysate partly restored smooth muscle content and decreased fibrosis in the corpus cavernosum, most importantly, neuronal nitric oxide. After these pioneering studies, several preclinical researchers evaluated the efficacy of SCs in rat model of nerve-sparing prostatectomy with positive results [75].

To progress toward clinical translation, Yiou et al. [76], in phase 1 trial, tested the efficacy of intracavernous injection of autologous BMSCs in 12 patients with post-prostatectomy ED. The patients were divided into four groups treated with escalating doses of BMSCs [76].

Only 4 of 12 men had nerve-sparing surgery. Patients reported subjective improvement in the Erectile Hardness Score. Doppler ultrasound showed an improvement when combined with pharmacotherapy. Importantly, no significant adverse reactions were reported after 6 months [76].

A similar clinical study using adipose stromal vascular fraction (SVF) [77] was conducted by Haahr et al. The adipose SVF provides a rich source of ADSCs and can easily be isolated from human adipose tissue, representing a viable alternative to bone marrow mesenchymal stem cells [77]. The SVF can be easily isolated through enzymatic digestion of aspirated adipose tissue, and it does not need to be cultured. Seventeen subjects suffering from ED after RP were enrolled. Adipose tissue was collected by liposuction under general anaesthesia and immediately processed (Celution 800/CRS system, Cytori Therapeutics, San Diego, CA, USA). Five patients reported minor events (Clavien-Dindo grade I) related mostly to the liposuction procedure. During the follow-up of this study, 8 of 17 patients engaged in successful sexual intercourse [77].

Further, large randomized studies are necessary to prove the real efficacy of stem cells treatment for ED after RP.

## Conclusion

Prostate cancer treatment has continued to progress resulting in favourable oncological outcomes. In this context, the quality of life of these patients, undergoing radical prostatectomy, has become of primary importance as part of their cancer survivorship. Although satisfactory results have been achieved for post-RP urinary continence, the same goals have not been replicated for erectile dysfunction after RP.

In this context, clinicians should be aware of the correct approaches to assist post-RP EF recovery.

Pathways to prevent post-RP ED clearly encompass all steps of the comprehensive clinical management of every PCa patient, including pre-operative, intraoperative and post-operative settings. Indeed, candidates for various surgical strategies should be carefully selected according to the baseline oncological and functional factors. Moreover, the advent of minimally invasive RP procedures has led to improved general anatomic knowledge and to the development of more conservative NS surgical techniques, thus facilitating a significant overall improvement in functional post-operative outcome.

Considering the failure of penile rehabilitation and the lack of evidence for APA preservation and nerve graft, nerve-sparing surgery and penile prostheses represent, today, the only methods to permanently and definitively preserve erectile function after RP.

## References

[1] Filson CP, Marks LS, Litwin MS (2015). Expectant management for men with early stage prostate cancer. CA Cancer J Clin.

[2] Nguyen LN, Head L, Witiuk K, Punjani N, Mallick R, Cnossen S, et al. The risks and benefits of cavernous neurovascular bundle sparing during radical prostatectomy: a systematic review and meta-analysis. J Urol. 2017. 10.1016/j.juro.2017.02.3344. 10.1016/j.juro.2017.02.3344 28286069

[3] Capogrosso P, Salonia A, Briganti A, Montorsi F (2016). Postprostatectomy erectile dysfunction: a review. World J Mens Health.

[4] Salonia A, Castagna G, Capogrosso P, Castiglione F, Briganti A, Montorsi F (2015). Prevention and management of post prostatectomy erectile dysfunction. Transl Androl Urol.

[5] Haglind E, Carlsson S, Stranne J, LAPPRO steering committee (2015). Urinary incontinence and erectile dysfunction after robotic versus open radical prostatectomy: a prospective, controlled, nonrandomised trial. Eur Urol.

[6] Fode M, Ohl DA, Ralph D, Sønksen J (2013). Penile rehabilitation after radical prostatectomy: what the evidence really says. BJU Int.

[7] Teloken P, Mesquita G, Montorsi F, Mulhall J (2009). Post-radical prostatectomy pharmacological penile rehabilitation: practice patterns among the international society for sexual medicine practitioners. J Sex Med.

[8] Dubbelman YD, Dohle GR, Schröder FH (2006). Sexual function before and after radical retropubic prostatectomy: a systematic review of prognostic indicators for a successful outcome. Eur Urol.

[9] Ficarra V, Novara G, Artibani W, Cestari A, Galfano A, Graefen M (2009). Retropubic, laparoscopic, and robot-assisted radical prostatectomy: a systematic review and cumulative analysis of comparative studies. Eur Urol.

[10] Ficarra V, Novara G, Ahlering TE, Costello A, Eastham JA, Graefen M (2012). Systematic review and meta-analysis of studies reporting potency rates after robot-assisted radical prostatectomy. Eur Urol.

[11] Gratzke C, Angulo J, Chitaley K, Dai YT, Kim NN, Paick JS (2010). Anatomy, physiology, and pathophysiology of erectile dysfunction. J Sex Med.

[12] John H, Hauri D (2000). Seminal vesicle-sparing radical prostatectomy: a novel concept to restore early urinary continence. Urology.

[13] Walz J, Epstein JI, Ganzer R, Graefen M, Guazzoni G, Kaouk J (2016). A critical analysis of the current knowledge of surgical anatomy of the prostate related to optimisation of cancer control and preservation of continence and erection in candidates for radical prostatectomy: an update. Eur Urol.

[14] Alsaid B, Bessede T, Diallo D, Moszkowicz D, Karam I, Benoit G (2011). Division of autonomic nerves within the neurovascular bundles distally into corpora cavernosa and corpus spongiosum components: immunohistochemical confirmation with three-dimensional reconstruction. Eur Urol.

[15] Walsh PC (2007). The discovery of the cavernous nerves and development of nerve sparing radical retropubic prostatectomy. J Urol.

[16] Gandaglia G, Lista G, Fossati N, Suardi N, Gallina A, Moschini M (2016). Non-surgically related causes of erectile dysfunction after bilateral nerve-sparing radical prostatectomy. Prostate Cancer Prostatic Dis.

[17] Salonia A, Burnett AL, Graefen M, Hatzimouratidis K, Montorsi F, Mulhall JP (2012). Prevention and management of postprostatectomy sexual dysfunctions part 2: recovery and preservation of erectile function, sexual desire, and orgasmic function. Eur Urol.

[18] Salonia A, Burnett AL, Graefen M, Hatzimouratidis K, Montorsi F, Mulhall JP, Stief C (2012). Prevention and management of postprostatectomy sexual dysfunctions part 1: choosing the right patient at the right time for the right surgery. Eur Urol.

[19] Weyne E, Castiglione F, Van der Aa F, Bivalacqua TJ, Albersen M (2015). Landmarks in erectile function recovery after radical prostatectomy. Nat Rev Urol.

[20] Weyne E, Mulhall J, Albersen M (2015). Molecular pathophysiology of cavernous nerve injury and identification of strategies for nerve function recovery after radical prostatectomy. Curr Drug Targets.

[21] Lue TF (2000). Erectile dysfunction. N Engl J Med.

[22] Sopko NA, Burnett AL (2016). Erection rehabilitation following prostatectomy—current strategies and future directions. Nat Rev Urol.

[23] Mulhall JP, Secin FP, Guillonneau B (2008). Artery sparing radical prostatectomy—myth or reality?. J Urol.

[24] Martínez-Salamanca JI, La Fuente JM, Martínez-Salamanca E, Fernández A, Pepe-Cardoso AJ, Louro N, Carballido J, Angulo J (2016). α_1A_-Adrenergic receptor antagonism improves erectile and cavernosal responses in rats with cavernous nerve injury and enhances neurogenic responses in human corpus cavernosum from patients with erectile dysfunction secondary to radical prostatectomy. J Sex Med.

[25] Bilhim T, Pisco JM, Rio Tinto H, Fernandes L, Pinheiro LC, Furtado A (2012). Prostatic arterial supply: anatomic and imaging findings relevant for selective arterial embolization. J Vasc Interv Radiol.

[26] Patel VR, Schatloff O, Chauhan S, Sivaraman A, Valero R, Coelho RF (2012). The role of the prostatic vasculature as a landmark for nerve sparing during robot-assisted radical prostatectomy. Eur Urol.

[27] Secin FP, Touijer K, Mulhall J, Guillonneau B (2007). Anatomy and preservation of accessory pudendal arteries in laparoscopic radical prostatectomy. Eur Urol.

[28] Henry BM, Pękala PA, Vikse J, Sanna B, Skinningsrud B, Saganiak K (2017). Variations in the arterial blood supply to the penis and the accessory pudendal artery: a meta-analysis and review of implications in radical prostatectomy. J Urol.

[29] Box GN, Kaplan AG, Rodriguez E, Skarecky DW, Osann KE, Finley DS (2010). Sacrifice of accessory pudendal arteries in normally potent men during robot-assisted radical prostatectomy does not impact potency. J Sex Med.

[30] Walz J, Burnett AL, Costello AJ, Eastham JA, Graefen M, Guillonneau B (2010). A critical analysis of the current knowledge of surgical anatomy related to optimization of cancer control and preservation of continence and erection in candidates for radical prostatectomy. Eur Urol.

[31] Montorsi F, Wilson TG, Rosen RC, Ahlering TE, Artibani W, Carroll PR (2012). Best practices in robot-assisted radical prostatectomy: recommendations of the pasadena consensus panel. Eur Urol.

[32] Tewari AK, Srivastava A, Huang MW, Robinson BD, Shevchuk MM, Durand M (2011). Anatomical grades of nerve sparing: a risk-stratified approach to neural-hammock sparing during robot-assisted radical prostatectomy (RARP). BJU Int.

[33] Schatloff O, Chauhan S, Sivaraman A, Kameh D, Palmer KJ, Patel VR (2012). Anatomic grading of nerve sparing during robot-assisted radical prostatectomy. Eur Urol.

[34] Tewari A, Srivasatava A, Menon M, Members of the VIP Team (2003). A prospective comparison of radical retropubic and robot-assisted prostatectomy: experience in one institution. BJU Int.

[35] Stolzenburg JU, Graefen M, Kriegel C, Michl U, Martin Morales A, Pommerville PJ (2015). Effect of surgical approach on erectile function recovery following bilateral nerve-sparing radical prostatectomy: an evaluation utilising data from a randomised, double-blind, double-dummy multicentre trial of tadalafil vs placebo. BJU Int.

[36] Ficarra V, Novara G, Fracalanza S, D’Elia C, Secco S, Iafrate M (2009). A prospective, non-randomized trial comparing robot-assisted laparoscopic and retropubic radical prostatectomy in one European institution. BJU Int.

[37] O’Neil B, Koyama T, Alvarez J, Conwill RM, Albertsen PC, Cooperberg MR (2016). The comparative harms of open and robotic prostatectomy in population based samples. J Urol.

[38] Alemozaffar M, Sanda M, Yecies D, Mucci LA, Stampfer MJ, Kenfield SA (2015). Benchmarks for operative outcomes of robotic and open radical prostatectomy: results from the health professionals follow-up study. Eur Urol.

[39] Ong WL, Evans SM, Spelman T, Kearns PA, Murphy DG, Millar JL (2015). Comparison of oncological and health related quality of life (HRQOL) outcomes between open (ORP) and robotic-assisted radical prostatectomy (RARP) for localized prostate cancer—findings from the population-based Victorian Prostate Cancer Registry (PCR). BJU Int.

[40] EAU Guidelines. Edn. presented at the EAU Annual Congress London 2017. ISBN 978–90–79754-91-5.

[41] Levine LA, Becher E, Bella A, Brant W, Kohler T, Martinez-Salamanca JI, Trost L, Morey A (2016). Penile prosthesis surgery: current recommendations from the International Consultation on Sexual Medicine. J Sex Med.

[42] Hellstrom WJ, Montague DK, Moncada I, Carson C, Minhas S, Faria G (2010). Implants, mechanical devices, and vascular surgery for erectile dysfunction. J Sex Med.

[43] Menard J, Tremeaux JC, Faix A, Pierrevelcin J, Staerman F (2011). Erectile function and sexual satisfaction before and after penile prosthesis implantation in radical prostatectomy patients: a comparison with patients with vasculogenic erectile dysfunction. J Sex Med.

[44] Bettocchi C, Palumbo F, Spilotros M, Lucarelli G, Palazzo S, Battaglia M (2010). Patient and partner satisfaction after AMS inflatable penile prosthesis implant. J Sex Med.

[45] Choi HM, Choi HK, Lee HY (2016). Urinary incontinence could be controlled by an inflatable penile prosthesis. World J Mens Health.

[46] Yiou R, Binhas M (2015). Combined implantation of a penile prosthesis and adjustable continence therapy ProACT in patients with erectile dysfunction and urinary incontinence after radical prostatectomy: results of a prospective pilot study. J Sex Med.

[47] Bolat D, Kozacioglu Z, Polat S, Koras O, Arslan M, Minareci S (2017). Synchronous penoscrotal implantation of penile prosthesis and artificial urinary sphincter after radical prostatectomy. Arch Esp Urol.

[48] Martínez-Salamanca JI, Espinós EL, Moncada I, Portillo LD, Carballido J (2015). Management of end-stage erectile dysfunction and stress urinary incontinence after radical prostatectomy by simultaneous dual implantation using a single trans-scrotal incision: surgical technique and outcomes. Asian J Androl.

[49] Tal R, Jacks LM, Elkin E, Mulhall JP (2011). Penile implant utilization following treatment for prostate cancer: analysis of the SEER-Medicare database. J Sex Med.

[50] Stephenson RA, Mori M, Hsieh YC, Beer TM, Stanford JL, Gilliland FD (2005). Treatment of erectile dysfunction following therapy for clinically localized prostate cancer: patient reported use and outcomes from the Surveillance, Epidemiology, and End Results Prostate Cancer Outcomes Study. J Urol.

[51] Bessede T, Moszkowicz D, Alsaid B, Zaitouna M, Diallo D, Peschaud F (2015). Inside-out autologous vein grafts fail to restore erectile function in a rat model of cavernous nerve crush injury after nerve-sparing prostatectomy. Int J Impot Res.

[52] Hu W, Cheng B, Liu T, Li S, Tian Y (2010). Erectile function restoration after repair of excised cavernous nerves by autologous vein graft in rats. J Sex Med.

[53] Quinlan DM, Nelson RJ, Walsh PC (1991). Cavernous nerve grafts restore erectile function in denervated rats. J Urol.

[54] Davis JW, Chang DW, Chevray P (2009). Randomized phase II trial evaluation of erectile function after attempted unilateral cavernous nerve-sparing retropubic radical prostatectomy with versus without unilateral sural nerve grafting for clinically localized prostate cancer. Eur Urol.

[55] Ogaya-Pinies G, Palayapalam-Ganapathi H, Rogers T, Hernandez-Cardona E, Rocco B, Coelho RF, et al. Can dehydrated human amnion/chorion membrane accelerate the return to potency after a nerve-sparing robotic-assisted radical prostatectomy? Propensity score-matched analysis. J Robot Surg. 2017. 10.1007/s11701-017-0719-8. 10.1007/s11701-017-0719-8 28656504

[56] Siddiqui KM, Billia M, Mazzola CR, Alzahrani A, Brock GB, Scilley C (2014). Three-year outcomes of recovery of erectile function after open radical prostatectomy with sural nerve grafting. J Sex Med.

[57] Rabbani F, Ramasamy R, Patel MI, Cozzi P, Disa JJ, Cordeiro PG (2010). Predictors of recovery of erectile function after unilateral cavernous nerve graft reconstruction at radical retropubic prostatectomy. J Sex Med.

[58] Satkunasivam R, Appu S, Al-Azab R, Hersey K, Lockwood G, Lipa J (2009). Recovery of erectile function after unilateral and bilateral cavernous nerve interposition grafting during radical pelvic surgery. J Urol.

[59] Hanson GR, Borden LS, Backous DD, Bayles SW, Corman JM (2008). Erectile function following unilateral cavernosal nerve replacement. Can J Urol.

[60] Fujioka M, Tasaki I, Kitamura R, Yakabe A, Hayashi M, Matsuya (2007). Cavernous nerve graft reconstruction using an autologous nerve guide to restore potency. BJU Int.

[61] Joffe R, Klotz LH (2007). Results of unilateral genitofemoral nerve grafts with contralateral nerve sparing during radical prostatectomy. Urology.

[62] Namiki S, Saito S, Nakagawa H, Sanada T, Yamada A, Arai Y (2007). Impact of unilateral sural nerve graft on recovery of potency and continence following radical prostatectomy: 3-year longitudinal study. J Urol.

[63] Saito S, Namiki S, Numahata K, Satoh M, Ishidoya S, Ito A (2007). Impact of unilateral interposition sural nerve graft on the recovery of sexual function after radical prostatectomy in Japanese men: a preliminary study. Int J Urol.

[64] Secin FP, Koppie TM, Scardino PT, Eastham JA, Patel M, Bianco FJ (2007). Bilateral cavernous nerve interposition grafting during radical retropubic prostatectomy: Memorial Sloan-Kettering Cancer Center experience. J Urol.

[65] Soebadi MA, Milenkovic U, Weyne E, Castiglione F, Albersen M (2017). Stem cells in male sexual dysfunction: are we getting somewhere?. Sex Med Rev.

[66] Soebadi MA, Moris L, Castiglione F, Weyne E, Albersen M (2016). Advances in stem cell research for the treatment of male sexual dysfunctions. Curr Opin Urol.

[67] Albersen M, Lin C-S, Lue T (2013). Stem-cell therapy for erectile dysfunction. Arab J Urol.

[68] Jankowski RJ, Deasy BM, Huard J (2002). Muscle-derived stem cells. Gene Ther.

[69] Caplan AI, Correa D (2011). The MSC: an injury drugstore. Cell Stem Cell.

[70] Usunier B, Benderitter M, Tamarat R, Chapel A (2014). Management of fibrosis: the mesenchymal stromal cells breakthrough. Stem Cells Int.

[71] Castiglione F, Hedlund P, Van der Aa F (2013). Intratunical injection of human adipose tissue-derived stem cells prevents fibrosis and is associated with improved erectile function in a rat model of Peyronie’s disease. Eur Urol.

[72] Castiglione F, Dewulf K, Hakim L, Weyne E, Montorsi F, Russo A (2016). Adipose-derived stem cells counteract urethral stricture formation in rats. Eur Urol.

[73] Bochinski D, Lin GT, Nunes L (2004). The effect of neural embryonic stem cell therapy in a rat model of cavernosal nerve injury. BJU Int.

[74] Kendirci M, Trost L, Bakondi B (2010). Transplantation of nonhematopoietic adult bone marrow stem/progenitor cells isolated by p75 nerve growth factor receptor into the penis rescues erectile function in a rat model of cavernous nerve injury. J Urol.

[75] Albersen M, Fandel TM, Lin G (2010). Injections of adipose tissue-derived stem cells and stem cell lysate improve recovery of erectile function in a rat model of cavernous nerve injury. J Sex Med.

[76] Yiou R, Hamidou L, Birebent B (2016). Safety of intracavernous bone marrow-mononuclear cells for postradical prostatectomy erectile dysfunction: an open dose-escalation pilot study. Eur Urol.

[77] Haahr MK, Jensen CH, Toyserkani NM (2016). Safety and potential effect of a single intracavernous injection of autologous adipose-derived regenerative cells in patients with erectile dysfunction following radical prostatectomy: an open-label phase I clinical trial. EBioMedicine.

